# Supplementary material for: Causal Relationship between Aging and Anorexia Nervosa: A White-Matter-Microstructure-Mediated Mendelian Randomization Analysis
Source: Biomedicines. 2024 Aug 16;12(8):1874. doi: 10.3390/biomedicines12081874 (PMC11351342; doi:10.3390/biomedicines12081874)
Supplement: Supplementary file 1 [file biomedicines-12-01874-s001.zip › Supplementary Figure/Figure S4/Singe SNP plot of MR analysis for TL to ubm-b-1457.pdf]

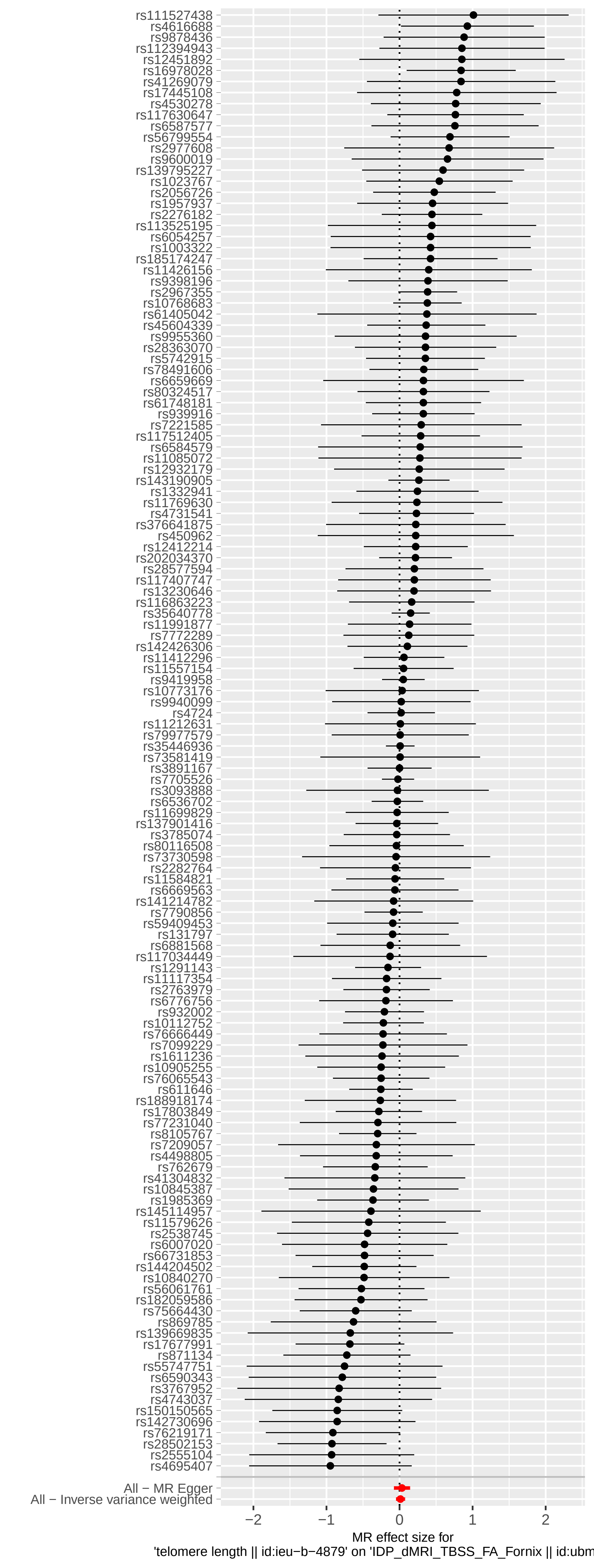
rs111527438
rs4616688
rs9878436
rs112394943
rs12451892
rs16978028
rs41269079
rs17445108
rs4530278
rs117630647
rs6587577
rs56799554
rs2977608
rs9600019
rs139795227
rs1023767
rs2056726
rs1957937
rs2276182
rs113525195
rs6054257
rs1003322
rs185174247
rs11426156
rs9398196
rs2967355
rs10768683
rs61405042
rs45604339
rs9955360
rs28363070
rs5742915
rs78491606
rs6659669
rs80324517
rs61748181
rs939916
rs7221585
rs117512405
rs6584579
rs11085072
rs12932179
rs143190905
rs1332941
rs11769630
rs4731541
rs376641875
rs450962
rs12412214
rs202034370
rs28577594
rs117407747
rs13230646
rs116863223
rs35640778
rs11991877
rs7772289
rs142426306
rs11412296
rs11557154
rs9419958
rs10773176
rs9940099
rs4724
rs11212631
rs79977579
rs35446936
rs73581419
rs3891167
rs7705526
rs3093888
rs6536702
rs11699829
rs137901416
rs3785074
rs80116508
rs73730598
rs2282764
rs11584821
rs6669563
rs141214782
rs7790856
rs59409453
rs131797
rs6881568
rs117034449
rs1291143
rs11117354
rs2763979
rs6776756
rs932002
rs10112752
rs76666449
rs7099229
rs1611236
rs10905255
rs76065543
rs611646
rs188918174
rs17803849
rs77231040
rs8105767
rs7209057
rs4498805
rs762679
rs41304832
rs10845387
rs1985369
rs145114957
rs11579626
rs2538745
rs6007020
rs66731853
rs144204502
rs10840270
rs56061761
rs182059586
rs75664430
rs869785
rs139669835
rs17677991
rs871134
rs55747751
rs6590343
rs3767952
rs4743037
rs150150565
rs142730696
rs76219171
rs28502153
rs2555104
rs4695407
All − MR Egger
All − Inverse variance weighted
−2
−1
0
1
2
MR effect size for
'telomere length || id:ieu−b−4879' on 'IDP_dMRI_TBSS_FA_Fornix || id:ubm
